# Vascular endothelial PDPK1 plays a pivotal role in the maintenance of pancreatic beta cell mass and function in adult male mice

**DOI:** 10.1007/s00125-019-4878-1

**Published:** 2019-05-04

**Authors:** Atsushi Obata, Tomohiko Kimura, Yoshiyuki Obata, Masashi Shimoda, Tomoe Kinoshita, Kenji Kohara, Seizo Okauchi, Hidenori Hirukawa, Shinji Kamei, Shuhei Nakanishi, Tomoatsu Mune, Kohei Kaku, Hideaki Kaneto

**Affiliations:** Department of Diabetes, Endocrinology and Metabolism, Kawasaki Medical School, 577 Matsushima, Kurashiki, 701-0192 Japan

**Keywords:** 3-Phosphoinositide-dependent protein kinase-1 (PDPK1), Capillary structure, Endothelial cells, Hypoxia, Pancreatic beta cells, Vascularity

## Abstract

**Aims/hypothesis:**

The aim of this study was to elucidate the impact of 3′-phosphoinositide-dependent protein kinase-1 (PDPK1) in vascular endothelial cells on the maintenance of pancreatic beta cell mass and function.

**Methods:**

Male vascular endothelial cell-specific *Pdpk1*-knockout mice (*Tie2*^+/−^/*Pdpk1*^flox/flox^ mice) and their wild-type littermates (*Tie2*^−/−^/*Pdpk1*^flox/flox^ mice; control) were used for this study. At 12 weeks of age, an IPGTT and OGTT were conducted. Pancreatic blood flow was measured under anaesthesia. Thereafter, islet blood flow was measured by the microsphere method. Mice were killed for islet isolation and further functional study and mRNA was extracted from islets. Pancreases were sampled for immunohistochemical analyses.

**Results:**

During the IPGTT, the blood glucose level was comparable between knockout mice and control *flox* mice, although serum insulin level was significantly lower in knockout mice. During the OGTT, glucose tolerance deteriorated slightly in knockout mice, accompanied by a decreased serum insulin level. During an IPGTT after pre-treatment with exendin-4 (Ex-4), glucose tolerance was significantly impaired in knockout mice. In fact, glucose-stimulated insulin secretion of isolated islets from knockout mice was significantly reduced compared with control *flox* mice, and addition of Ex-4 revealed impaired sensitivity to incretin hormones in islets of knockout mice. In immunohistochemical analyses, both alpha and beta cell masses were significantly reduced in knockout mice. In addition, the CD31-positive area was significantly decreased in islets of knockout mice. The proportion of pimonidazole-positive islets was significantly increased in knockout mice. mRNA expression levels related to insulin biosynthesis (*Ins1*, *Ins2*, *Mafa*, *Pdx1* and *Neurod* [also known as *Neurod1*]) and beta cell function (such as *Gck* and *Slc2a2*) were significantly decreased in islets of knockout mice. Microsphere experiments revealed remarkably reduced islet blood flow. In addition, mRNA expression levels of *Hif1α* (also known as *Hif1a*) and its downstream factors such as *Adm*, *Eno1*, *Tpi1* (also known as *Ets1*), *Hmox1* and *Vegfa*, were significantly increased in islets of knockout mice, indicating that islets of knockout mice were in a more hypoxic state than those of control *flox* mice. As a result, mRNA expression levels related to adaptive unfolded protein response and endoplasmic reticulum stress-related apoptotic genes were significantly elevated in islets of knockout mice. In addition, inflammatory cytokine levels were increased in islets of knockout mice. Electron microscopy revealed reduced endothelial fenestration and thickening of basal membrane of vascular endothelium in islets of knockout mice.

**Conclusions/interpretation:**

Vascular endothelial PDPK1 plays an important role in the maintenance of pancreatic beta cell mass and function by maintaining vascularity of pancreas and islets and protecting them from hypoxia, hypoxia-related endoplasmic reticulum stress, inflammation and distortion of capillary structure.

**Electronic supplementary material:**

The online version of this article (10.1007/s00125-019-4878-1) contains peer-reviewed but unedited supplementary material, which is available to authorised users.

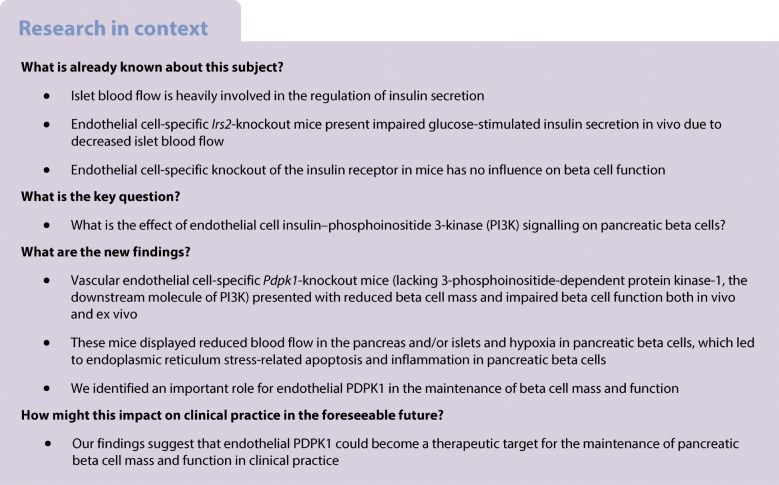



## Introduction

Diabetes is the most prevalent and serious metabolic disease and the number of diabetic individuals has been increasing markedly all over the world. The hallmark of the disease is insulin resistance and pancreatic beta cell dysfunction. Under diabetic conditions, insulin target tissues, such as liver, muscle and fat, become resistant to insulin and pancreatic beta cell function gradually deteriorates [1–3]. Beta cell failure and/or dysfunction is strongly associated with type 2 diabetes and may lead to the onset of hyperglycaemia. Therefore, amelioration of impaired insulin secretion might be a prospective therapeutic goal.

Several studies have reported that pancreatic islets are densely vascularised and many mediators, such as insulin, regulate the islet blood flow, which is highly involved in the regulation of insulin secretion [4, 5].

Kondo et al reported that vascular endothelial cell-specific insulin receptor knockout (VENIRKO) or insulin like growth factor-1 receptor knockout (VENIFARKO) resulted in reduced angiogenesis of the retina [6]. However, these models did not present insulin resistance. Mukai et al reported that endothelial cell-specific constitutive activation of *Akt* suppresses vascular lesion formation via increased NO production, preservation of functional endothelial layer and suppression of inflammatory and proliferative changes in the vascular wall [7]. Indeed, these studies elucidated the role played by insulin–phosphoinositide 3-kinase (PI3K) signalling in endothelial cells. However, neither of the studies conducted assessment on pancreatic beta cells.

Recently, it was reported that endothelial cell-specific insulin receptor knockout (EndoIRKO) mice presented glucose intolerance and insulin resistance, which was a different phenotype from those used by Kondo et al, probably for the following reasons. First, the mice were backcrossed to C57BL/6, and VE-*Cadherin* Cre mice were used instead of *Tie-2* Cre mice. Second, the mice presented as insulin resistant because of delayed insulin delivery to systemic organs, except for the liver and olfactory bulb in which the fenestrated endothelium of the capillaries freely permits paracellular passage of macromolecules [8]. In this model, no morphological and functional changes in the islets were observed. On the contrary, endothelial cell-specific *Irs2*-knockout (ETIRS2KO) mice also showed insulin resistance mainly in skeletal muscle due to reduced capillary recruitment [9]. Intriguingly, reduced glucose-stimulated insulin secretion (GSIS) in vivo due to decreased islet blood flow was also observed in this model without any morphological changes in islets [10]. As seen in these previous reports, many things still remain to be unravelled about the influence of insulin–PI3K signalling of endothelial cells on pancreatic beta cells.

Therefore, in the present study, we focused on 3′-phosphoinositide-dependent protein kinase-1 (PDPK1), a serine-threonine kinase that mediates downstream signalling of PI3K and regulates the activity of *Akt*, to further elucidate the impact of insulin–PI3K signalling in endothelial cells on pancreatic beta cells. We investigated the role played by endothelial PDPK1 in the maintenance of pancreatic beta cell mass and function using vascular endothelial cell-specific *Pdpk1*-knockout mice.

## Methods

### Vascular endothelial cell-specific *Pdpk1*-knockout mice

Mice were kindly provided by K. Kotani (Saso Hospital, Kobe, Japan), W. Ogawa (Division of Diabetes, Metabolism, and Endocrinology, Kobe University Graduate School of Medicine, Kobe, Japan) and M. Kasuga (Research Institute, International Medical Center of Japan, Tokyo, Japan). As described previously [11], vascular endothelial cell-specific *Pdpk1*-knockout (VE-PDPK1-KO) mice were generated by breeding *Pdpk1*^flox/flox^ mice, which harbour a modified endogenous *Pdpk1* gene in which exons 3 and 4 are flanked by loxP sites, with mice that express the *Cre* recombinase gene under the control of the *Tie2* gene promoter (*Tie2*-*Cre*) [12]. The heterozygous offspring of both the loxP-targeted *Pdpk1* gene and the *Tie2*-*Cre* transgene (*Tie2-Cre*^+/−^/*Pdpk1*^flox/+^) were then crossed with *Pdpk1*^flox/flox^ mice to generate VE-PDPK1-KO mice homozygous for the *Pdpk1* floxed allele (*Tie2-Cre*^+/−^/*Pdpk1*^flox/flox^). These mice were all bred from C57BL/6J mice. VE-PDPK1-KO mice were born at the expected Mendelian frequency and their littermates, which did not express *Tie2*-*Cre* (*Tie2-Cre*^−/−^/*Pdpk1*^flox/flox^), were used as control mice. Mice were housed in a 12 h dark–light cycle at a controlled temperature and were allowed free access to water and a standard diet. Male mice were used for all experiments at 12 weeks of age. All aspects of animal care and experiments were performed at the Laboratory Animal Center with the approval of the Animal Research Committee at the Kawasaki Medical School (No. 18-004).

### IPGTT and OGTT

After 16 h fasting, d-(+)-glucose (1.5 g/kg) was administered by i.p. injection for the IPGTT or administered orally for the oral glucose tolerance test (OGTT). Blood samples from tail snips were collected at the indicated time point and the blood glucose level was measured using a Free Style Kissei glucose meter (Kissei Pharmaceutical, Tokyo, Japan). For pre-treatment with exendin-4 (Ex-4) in the IPGTT, 100 ng of Ex-4 was given by i.p. injection 15 min before glucose injection. For measurements of active glucagon-like peptide-1 (GLP-1) and glucagon, EDTA and aprotinin were placed in sample collection tubes beforehand. Active GLP-1 level was measured using GLP-1, active form assay (IBL, Gunma, Japan). Glucagon level was determined using glucagon ELISA (Mercodia, Uppsala, Sweden). Serum insulin level was determined using a mouse insulin ELISA kit (Morinaga, Tokyo, Japan).

### Pancreatic islet isolation

Isolation of islets from the pancreas of the mice was conducted as previously described [13, 14]. In brief, after ligation of the common bile duct with silk thread at a point close to the duodenal outlet, 2.5 ml of Hanks’ Balanced Salt Solution (HBSS) (Sigma, St Louis, MO, USA) containing 0.6 mg Liberase TL (Roche Diagnostics, Tokyo, Japan) and 25 mmol/l HEPES were injected into the duct. The swollen pancreas was removed and incubated at 37°C for 24 min. The pancreatic tissue was then dispersed by pipetting and washed twice with ice-cold HBSS containing 25 mmol/l HEPES and 10% (wt/vol.) FBS. Thereafter, the islets were manually picked up under a stereoscopic microscope and used immediately for the experiments.

### GSIS from isolated pancreatic islets

Size-matched pancreatic islets were prepared (five pancreatic islets/tube) and pre-incubated in KRB–HEPES buffer. The supernatant fraction was replaced with glucose solution (either 3 mmol/l or 16.7 mmol/l) and the mixture was incubated for an additional 60 min at 37°C. The supernatant fraction was recovered and stored at −80°C until use. Thereafter, the solution which contained 16.7 mmol/l glucose and 10 nmol/l Ex-4 was added and incubated for another 60 min at 37°C. The supernatant fraction was collected and stored at −80°C until use.

### Islet perifusion

Islet perifusion in vitro was conducted as performed by Hashimoto et al with slight modification [10]. In our study, ten size-matched islets were prepared for analysis.

### Measurement of pancreatic and islet blood flow

Mice were anaesthetised with sevoflurane and the body temperature was kept at 37°C. Pancreatic blood flow was measured using MoorFLPI-2 laser-Doppler apparatus (Moor Instruments, Axminster, Devon, UK), in accordance with the manufacturer’s instruction, during the systolic phase. To further elucidate islet blood flow, a microsphere experiment was conducted as performed by Hashimoto et al [10] and Jansson and Hellerström [15], with slight modifications. Polyethylene catheters (SP10: Natsume Seisakusho, Tokyo, Japan) were inserted into the left carotid artery and the left femoral artery. In our study, Dye-Trak microspheres (Triton Technology, Los Angeles, CA, USA) with a mean diameter of 15 mm were injected for 15 s into the ascending aorta. Arterial blood sample was collected from the femoral artery at a constant rate of 0.4 ml/min by using a syringe pump, starting 5 s before the microsphere injection and continued for 60 s. The whole pancreas was removed, blotted, weighed and treated using a freeze–thawing technique to visualise the microspheres and the number of microspheres in islets was counted with a dark field microscope. After counting the number of microspheres, pancreatic tissue was collected again into a tube and the tissue was dissolved with 2% (vol./vol.) Tween80 + 4 mol/l KOH. After sonication, the sample was diluted ten times with PBS and the number of microspheres was counted on the 10 μm pore filter (Triton Technology) with a stereoscopic microscope. Alternatively, the collected blood sample was dissolved with 16 mol/l KOH + 2% (vol./vol.) Tween 80 and the number of microspheres was counted by using a haemocytometer. The blood flow values were calculated according to the following formula: organ blood flow = 0.4 (ml/min) × no. of microspheres in target organ / number of microspheres in blood / weight of target organ. The islet blood flow was expressed per islet weight estimated by multiplying the pancreatic weight with the islet volume fraction of the whole pancreas, which was evaluated by insulin and glucagon double staining.

### Histological and immunohistochemical analyses of islets

Isolated pancreases were fixed overnight with 4% (vol./vol.) paraformaldehyde at 4°C. Tissues were routinely processed for paraffin embedding and 4 μm sections were cut and mounted on silanised slides. The sections were incubated with a mixture of primary antibodies (sc-130624 glucagon antibody (79/bB10) mouse monoclonal IgG; diluted 1:100 by PBS supplemented with 1% (wt/vol.) BSA (1% BSA) and sc-9168 insulin antibody (H-86) rabbit polyclonal IgG; diluted 1:200 by 1% BSA; Santa Cruz Biotechnology, Santa Cruz, CA, USA). For secondary antibody, donkey anti-rabbit IgG (H+L) secondary antibody Alexa Fluor 488 conjugate (A-21206, Life Technologies, Carlsbad, CA, USA) diluted 1:1000 by 1% BSA and donkey anti-mouse IgG (H+L) secondary antibody Alexa Fluor 594 conjugate (A-21203, Life Technologies, Carlsbad, CA, USA) diluted 1:1000 by 1% BSA were applied. Pancreatic islet proliferation was identified by staining sections with rabbit anti-Ki-67 polyclonal antibody (Abcam, Cambridge, MA, USA). For primary antibodies, guinea pig polyclonal IgG anti-insulin antibody (ab7842; diluted 1:100 by 1% BSA) and rabbit polyclonal IgG anti-Ki67 antibody (ab15580; diluted 1:500 by 1% BSA) were used. For secondary antibody, goat anti-guinea pig IgG (H+L) secondary antibody Alexa Fluor 488 conjugate (A-11073, Life Technologies, Carlsbad, CA, USA) diluted 1:1000 by 1% BSA and donkey anti-rabbit IgG (H+L) secondary antibody Alexa Fluor 594 conjugate (A-21207, Life Technologies, Carlsbad, CA, USA) were applied. To investigate cell apoptosis in pancreatic islets, a TUNEL assay was performed using a DeadEnd Fluorometric TUNEL System (DeadEnd; cat. no. G3250, Promega, Madison, WI, USA) as previously reported [16]. For CD31 staining, isolated pancreases were embedded in Tissue-Tek Optimal Cutting Temperature compound (Funakoshi, Tokyo, Japan) and frozen by liquid nitrogen. Sections (10 μm) were cut and mounted on slides in a cryostat. The sections were incubated with primary antibody (purified rat anti-mouse CD31, cat. no. 550274; BD Pharmingen) and CD31 was detected by using Anti-Ig HRP Detection Kits (cat. no. 551013, BD Pharmingen, San Diego, CA, USA) in accordance with the manufacturer’s instruction followed by H&E counterstaining. Immunohistochemical staining for pimonidazole was conducted as performed by Hashimoto et al [10] and Takikawa et al [17], with slight modifications. Hypoxyprobe -1 Plus Kit (HP2-100, Hypoxyprobe, Burlington, MA, USA) was used in accordance with the manufacturer’s instruction. In our study, pancreases were collected 1 h after pimonidazole injection.

### Morphometric analysis

The image analysis software NIH Image (version 1.61; http://rsbweb.nih.gov/ij/) was used to calculate the pancreas area and islet area. Using a total of nine sections (three sections from three different areas of the pancreas) for each group of mice, beta cell mass was estimated via the following formula: cell mass (mg) = average of islet area per section / average of pancreas area per section × weight of pancreas × beta cell ratio (average of beta cell number / cell number in islet). Observations were made using a minimum of 50 islets and, when quantified, were expressed as a percentage of the total number of islet cells. CD31-positive area and vascular structure numbers/mm^2^ in islets were calculated using NIH Image (version 1.61; http://rsbweb.nih.gov/ij/).

### Electron microscopy

The islets were fixed in a solution of 2.5% (vol./vol.) glutaraldehyde soon after isolation. The samples were fixed for 2 h in 2.5% (vol./vol.) glutaraldehyde buffered to pH 7.4 with phosphate buffer and treated with osmium tetroxide for 1 h at 4°C. The tissues were dehydrated with graded concentration ethanol and then embedded in Epon 812 (Nakarai, Kyoto, Japan). Thin sections were cut with a Leica Ultracut S (Leica Microsystems, Tokyo, Japan) with a diamond knife and stained with uranyl acetate followed by lead citrate [18]. Electron micrographs were taken with a JEOL JEM-1400 electron microscope (JEOL, Tokyo, Japan) operated at 80 kV.

### RNA preparation and quantitative PCR

Total RNA was extracted using an RNeasy lipid tissue mini kit (Qiagen, Valencia, CA, USA) according to the manufacturer’s instructions. cDNA was produced from mRNA using TaqMan reverse transcription reagents (Applied Biosystems, Foster City, CA, USA). Quantitative PCR (qPCR) was performed using a 7500 Real-Time PCR system (Applied Biosystems). The relative expression levels were compared by normalisation to the expression levels of β-actin. The primers were purchased from Takara Bio Co. (Shiga, Japan); sequences are shown in ESM Table 1.

### Statistics

Results are expressed as mean ± SEM. Differences between two groups were tested for statistical significance using Student’s *t* test. Differences among more than two groups were tested using the Tukey–Kramer method. *p* values less than 0.05 were considered to denote statistical significance.

## Results

### Glucose stimulated insulin secretion was impaired in islets of VE-PDPK1-KO mice compared with control *flox* mice both in vivo and in vitro

**Fig. 1:**
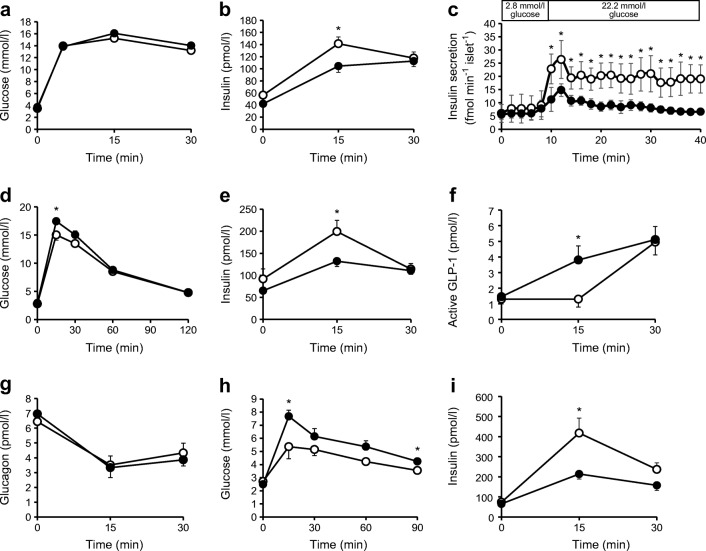
IPGTT and OGTT revealed lower insulin levels in VE-PDPK1-KO mice, and an isolated islet perifusion study revealed a deterioration in GSIS in knockout mice. (**a**, **b**) Blood glucose levels (**a**) and serum insulin levels (**b**) during an IPGTT (*n*=7 mice). (**c**) Islet perifusion experiment; *n*=5 (control *flox* mice) or *n*=4 (VE-PDPK1-KO mice). (**d**–**g**) Blood glucose, insulin, active GLP-1 and glucagon levels during an OGTT; *n*=7 (control *flox* mice) or *n*=8 (VE-PDPK1-KO mice) (**h**, **i**) IPGTT conducted after 100 ng Ex-4 pre-treatment; *n*=7 (control *flox* mice) or *n*=8 (VE-PDPK1-KO mice). Black circles, VE-PDPK1-KO mice; white circles, control *flox* mice. Values are means ± SEM. *p<0.05 vs control *flox* mice

IPGTT conducted when mice were 12 weeks old revealed lower insulin levels in VE-PDPK1-KO mice compared with control *flox* mice, although there was no significant difference in blood glucose levels (Fig. 1a,b). Next, the islet perifusion experiment in vitro was conducted to investigate GSIS. Both first and second phases of GSIS were impaired in islets of VE-PDPK1-KO mice after exposure to high-glucose KRB buffer (Fig. 1c).

In the OGTT, glucose tolerance was slightly but significantly deteriorated in VE-PDPK1-KO mice, accompanied by reduced serum insulin levels (Fig. 1d,e). Unexpectedly, during the OGTT, active GLP-1 levels were significantly increased in the knockout mice, although there was no difference in serum glucagon levels (Fig. 1f,g). These results suggested that sensitivity to incretin hormone was impaired in knockout mice. Therefore, an IPGTT was conducted after pre-treatment with Ex-4. This showed impaired glucose tolerance and reduced insulin levels in the knockout mice (Fig. 1h,i). These results suggested that sensitivity to incretin hormone was definitely impaired in VE-PDPK1-KO mice.

**Fig. 2:**
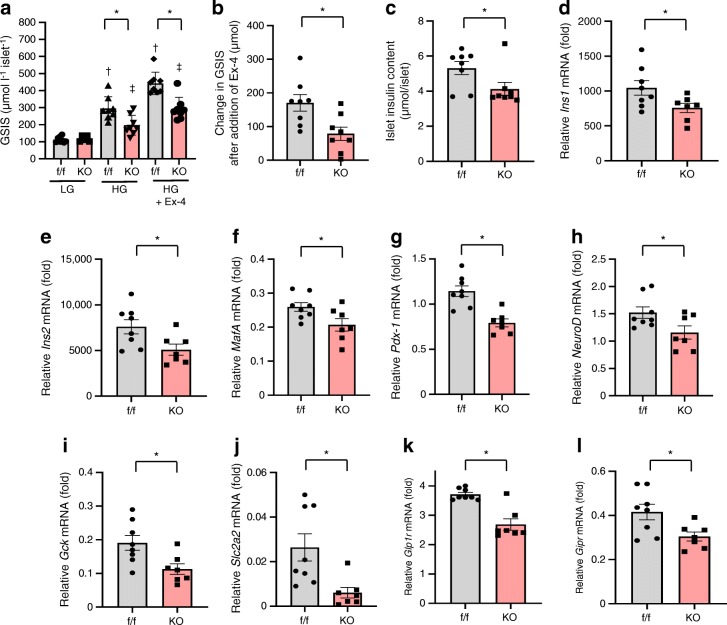
GSIS of isolated islets and mRNA expression levels related to insulin, its biosynthesis and beta cell function. (**a**) GSIS of isolated islets from control *flox* and VE-PDPK1-KO mice (*n*=8 mice), in 3.3 mmol/l glucose (low glucose, LG) and 16.7 mmol/l glucose (high glucose, HG), without and with addition of 10 nmol/l Ex-4. (**b**) Change in GSIS after addition of Ex-4 in HG (*n*=8 mice). (**c**) Islet insulin content (*n*=8 mice). (**d**–**l**) Expression levels of mRNA (reported as fold vs β-actin) related to insulin biosynthesis (**d**–**h**) and beta cell function (**i**–**l**) (*n*=7 or 8 mice, as indicated). Values are the means ± SEM. *p<0.05, KO vs f/f; ^†^*p*<0.05 vs LG f/f; ^‡^*p*<0.05 vs LG KO. f/f, control *flox* mice; HG, high glucose (16.7 mmol/l); KO, VE-PDPK1-KO mice; LG, low glucose (3.3 mmol/l)

To further investigate islet function, GSIS was conducted in vitro. GSIS was significantly impaired at high glucose concentrations (Fig. 2a). Moreover, addition of Ex-4 to high-glucose solution revealed a reduced reaction to Ex-4 in islets of VE-PDPK1-KO mice (Fig. 2b). Islet insulin content was significantly decreased in knockout mice (Fig. 2c). mRNA expression levels related to insulin biosynthesis, such as *Ins1*, *Ins2*, *Mafa*, *Pdx1* and *Neurod*, were all significantly reduced in VE-PDPK1-KO mice (Fig. 2d–h). In addition, mRNA expression levels related to GSIS, such as *Gck* and *Slc2a2*, were significantly decreased in the knockout mice (Fig. 2i,j). Furthermore, *Glp1r* expression levels were also significantly decreased in VE-PDPK1-KO mice, suggesting that there is reduced sensitivity to incretin hormone in knockout mice (Fig. 2k). As *Pdx1* and *Mafa* are essential for differentiation of pancreatic beta cells, we evaluated the mRNA expression level of *Ngn3*, a marker of endocrine precursor cells, to confirm whether differentiation of pancreatic beta cells was affected by endothelial cell-specific *Pdpk1* knockout. As shown in ESM Fig. 1, *Ngn3* mRNA expression level was comparable between control flox and knockout mice. Therefore, we think that beta cell differentiation was not affected in these mice but rather that beta cell de-differentiation was not facilitated by endothelial cell-specific *Pdpk1* knockout.

### Both pancreatic beta cell mass and alpha cell mass were reduced in VE-PDPK1-KO mice compared with control *flox* mice

**Fig. 3:**
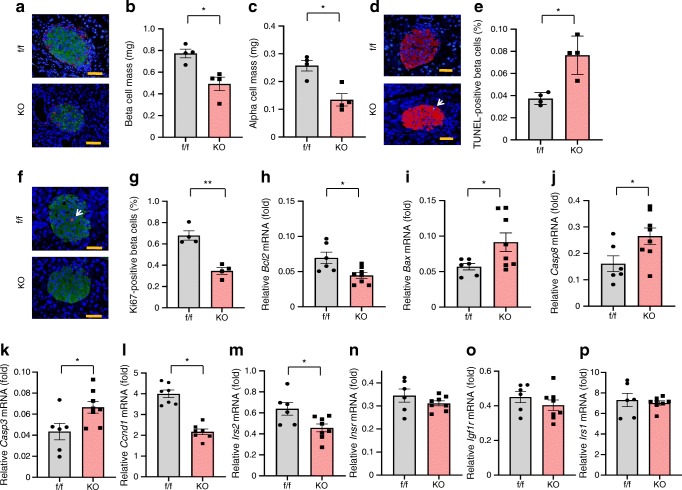
Evaluation of morphology of islets and mRNA expression levels related to beta cell proliferation, apoptosis and beta cell mass. (**a**) Representative images of insulin and glucagon double staining of pancreatic sections in control flox and VE-PDPK1-KO mice; scale bars, 50 μm; green, insulin; red, glucagon; blue, DAPI. (**b**, **c**) Quantification of beta (**b**) and alpha (**c**) cell mass (*n*=4 mice). (**d**) Representative images of TUNEL assay. White arrow indicates TUNEL-positive beta cell; scale bars, 50 μm; red, insulin; blue, DAPI. (**e**) TUNEL-positive beta cells were counted and divided by total beta cell number (*n*=4 mice). (**f**) Representative images of Ki67 staining. White arrow indicates Ki67-positive beta cell; scale bars, 50 μm; green, insulin; blue, DAPI. (**g**) Quantification of Ki67-positive beta cells (*n*=4 mice). (**h**–**p**) Expression levels of mRNA (reported as fold vs β-actin) related to apoptosis (**h**–**k**) and beta cell mass (**l**–**p**) (*n*=6 or 8 mice, as indicated). Values are the means ± SEM. *p<0.05, **p<0.01, KO vs f/f. f/f, control *flox* mice; KO, VE-PDPK1-KO mice

Next, the morphology of islets was investigated by insulin and glucagon double staining. This revealed significantly reduced beta cell mass and alpha cell mass in VE-PDPK1-KO mouse islets (Fig. 3a–c). In TUNEL assay, the proportion of TUNEL-positive nuclei was significantly higher in islets from knockout mice than in islets from control *flox* mice (Fig. 3d,e). In addition, the proportion of Ki-67-positive nuclei was significantly lower in knockout mouse islets (Fig. 3f,g). These results suggested that proliferation of beta cells was impaired and apoptosis of beta cells was increased in VE-PDPK1-KO mice. Corresponding to these immunohistochemical analyses, mRNA expression levels related to apoptosis such as *Bax*, *Casp8* and *Casp3* were significantly increased in VE-PDPK1-KO mice while the mRNA expression level of level of *Bcl2*, an anti-apoptotic gene, was significantly reduced (Fig. 3h–k). In addition, mRNA expression levels related to cell replication and proliferation, such as *Ccnd1* and *Irs2*, were significantly decreased in VE-PDPK1-KO mice (Fig. 3l–p). These results supported the notion that beta cell mass reduction was induced by increased apoptosis and decreased proliferation of beta cell in the knockout mice.

### Both pancreatic and islet blood flows were significantly reduced in VE-PDPK1-KO mice compared with control *flox* mice

**Fig. 4:**
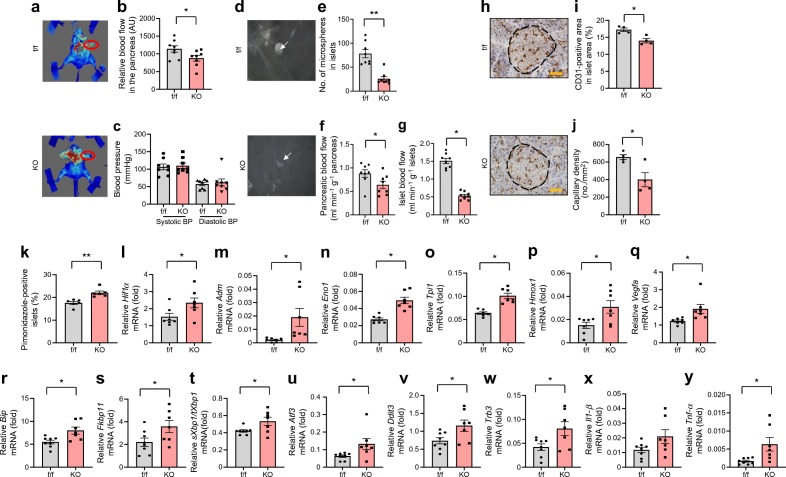
Pancreatic and islet blood flow, CD31 and pimonidazole staining and mRNA expression levels related to hypoxia, ER stress and inflammation. (**a**) Representative images of the laser-Doppler experiment for evaluation of pancreatic blood flow. The pancreas is indicated by a red circle. The intensity of the red colour was measured using MoorFLPI-2 apparatus. (**b**) Pancreatic blood flow, shown as arbitrary units (AU) (*n*=8 mice). (**c**) Blood pressure (*n*=8 mice). (**d**–**g**) Representative images of darkfield microscopy (**d**). Islets are shown in white and the arrows point to microspheres in the islet, which appear as small black dots; the microspheres were counted to evaluate islet blood flow (**e**). Pancreatic (**f**) and islet (**g**) blood flow evaluated by microspheres (*n*=8 mice). (**h**–**j**) Representative images of CD31 staining (**h**). The black dotted line indicates an islet and brown staining indicates capillaries. The brown area was measured and divided by total islet area to give the CD31-positive area in islet area (%) (**i**), and the brown structures were counted and divided by total islet area to give capillary density (**j**) (*n*=4 mice); scale bars, 50 μm. (**k**) Percentage of pimonidazole-positive islets (*n*=6 mice). (**l**–**q**) Expression levels of *Hif1α* mRNA and its downstream factors (reported as fold vs β-actin) (*n*=7 mice). (**r**–**y**) Expression levels of mRNA (reported as fold vs β-actin except for **t**, in which expression level of *sXbp1* [fold vs β-actin] was divided by that of *tXbp1* [fold vs β-actin]) related to adaptive UPR and ER stress-related apoptotic genes (**r**–**w**) and inflammation (**x**, **y**) (*n*=7 or 8 mice, as indicated). Values are the means ± SEM. *p<0.05 and **p<0.01, KO vs f/f; in (**x**), *p*=0.075 for KO vs f/f. f/f, control *flox* mice; KO, VE-PDPK1-KO mice

Next, pancreatic blood flow was investigated. Intriguingly, pancreatic blood flow was significantly decreased in VE-PDPK1-KO mice, although blood pressure and pancreas weight were comparable between the knockout mice and their control *flox* mice (Fig. 4a–c, and ESM Fig. 2). Next, a microsphere experiment was conducted to precisely evaluate islet blood flow. The number of microspheres in islets was significantly decreased in VE-PDPK1-KO mice (Fig. 4d,e). Whole pancreatic blood flow, which was calculated by microsphere experiment, was comparable with that measured by laser-Doppler apparatus (Fig. 4f). Intriguingly, islet blood flow was drastically reduced in knockout mice (Fig. 4g). In addition, CD31-positive area and capillary density were significantly decreased in VE-PDPK1-KO mice (Fig. 4h–j). As a result, the percentage of pimonidazole-positive islets was significantly increased in VE-PDPK1-KO mice (Fig. 4k), suggesting that islets of the knockout mice were in a more hypoxic state compared with control *flox* mice. In fact, mRNA levels of *Hif1α* (also known as *Hif1a*) and its downstream genes *Adm*, *Eno1*, *Tpi1* (also known as *Ets1*), *Hmox1* and *Vegfa* were significantly increased in the knockout mice (Fig. 4l–q).

As islets of VE-PDPK1-KO mice were in a relatively more hypoxic state than those of control *flox* mice, we next investigated the endoplasmic reticulum (ER) stress-related adaptive unfolded protein response (UPR) and apoptotic gene expression levels. mRNA expression levels related to adaptive UPR, such as *Bip*, *Fkbp11* and *sXbp1/tXbp1*, were significantly increased in VE-PDPK1-KO mice (Fig. 4r–t). In addition, the expression levels of ER stress-related apoptotic genes, such as *Atf3*, *Ddit3* and *Trb3* (also known as *Trib3*), were all significantly elevated in knockout mice (Fig. 4u–w). Furthermore, expression levels of inflammatory cytokine genes, *Il1-β* (also known as *Il1b*) and *Tnf-α* (also known as *Tnf*), were increased in islets of knockout mice (Fig. 4x,y). These results suggest that hypoxia-induced ER stress and inflammation led to the decreased pancreatic beta cell mass and impairment of beta cell function.

**Fig. 5:**
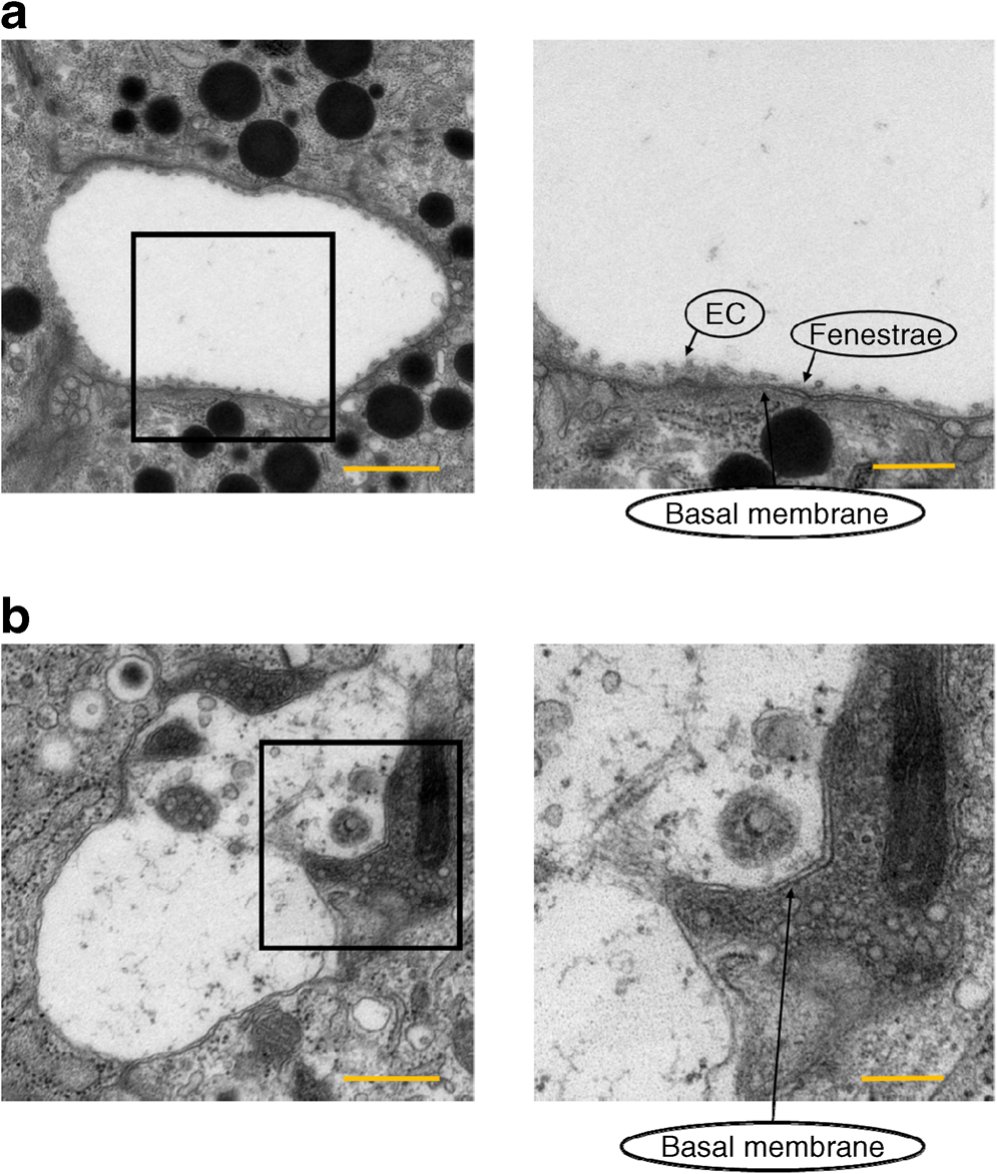
Vascular structure of islets from VE-PDPK1-KO mice and control *flox* mice. Electron microscopic images showing capillaries surrounded by pancreatic beta cells in (**a**) control *flox* mice and (**b**) VE-PDPK1-KO mice. EC, endothelial cells. Images are representative of four mice. Magnification ×5000 (left), scale bars, 1 μm; or ×10,000 (right), scale bars, 500 nm

Furthermore, electron microscope-elucidated vascular structure in islets was markedly altered in VE-PDPK1-KO mice (Fig. 5a,b). In control *flox* mice, endothelial cells were aligned along the basal membrane and they exhibited structured fenestrations (Fig. 5a). On the other hand, in VE-PDPK1-KO mice, there were less endothelial cells along basal membrane, so-called ‘empty sleeves’, and thickening of the basal membrane was observed (Fig. 5b). We also evaluated mRNA expression levels of angiocrine factors such as *Hgf* and *Fgf2*, and found no difference between control *flox* and VE-PDPK1-KO mice (ESM Fig. 3).

## Discussion

**Fig. 6:**
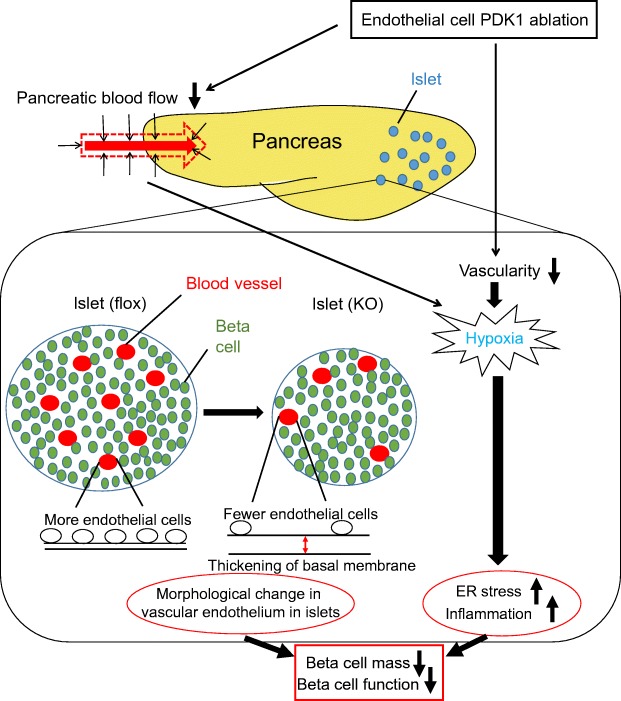
Possible mechanism of how endothelial cell PDPK1 maintains beta cell mass and function. Ablation of endothelial PDPK1 causes a reduction of vascularity in islets, and both pancreatic and islets blood flow is decreased, which leads to hypoxia in islets, which in turn induces ER stress-related apoptosis and inflammation. Furthermore, PDPK1 ablation in endothelial cells leads to distortion of capillary structure in islets. For example, there are fewer endothelial cells along the basal membrane, together with a thickening of the basal membrane. Thus, vascular endothelial PDPK1 plays an important role in the maintenance of pancreatic beta cell mass and function by maintaining the vascularity of the pancreas and islets and protecting them from hypoxia, hypoxia-related ER stress, inflammation and distortion of capillary structure

In the present study, we elucidated endothelial-specific ablation of *Pdpk1* impaired GSIS by reducing pancreatic and/or islet blood flow. We also demonstrate that these decreases in blood flow induce hypoxia in islets of VE-PDPK1-KO mice and that hypoxia-induced ER stress and inflammation lead to beta cell mass reduction and impairment of beta cell function accompanied by morphological changes of capillaries in islets (Fig. 6).

Our in vitro results suggest that GSIS is impaired (Fig. 1c, Fig. 2a,b). However, the perifusion experiment did not end with a period of low glucose and we did not confirm that the insulin secretion decreased again during low glucose, so we cannot completely exclude the possibility that the preparation was damaged to some extent during the experiment.

In our study, OGTT revealed unexpectedly elevated levels of active GLP-1 in VE-PDPK1-KO mice (Fig. 1f). This might be a compensational elevation due to chronically impaired sensitivity to incretin hormone in islets of the knockout mice. The *Glp1r* mRNA level was significantly reduced in knockout mice. However, the level of GLP-1 receptor protein was not assessed by western blotting or immunostaining in this study as there was no appropriate antibody commercially available. This could be a limitation of our study. In addition, serum glucagon level was not decreased during OGTT in VE-PDPK1-KO mice although insulin and glucagon double staining revealed decreased alpha cell mass (Fig. 1g, Fig. 3c). This might be explained by the phenomena that blood glucose level was maintained within physiological range, and not hypoglycaemic range. Hyperinsulinaemic–hypoglycaemic clamp might reveal the difference in glucagon levels and needs further investigation to address this question.

Several studies have demonstrated that islets in animal models of diabetes, such as *db/db* mice, KKay mice and Zucker diabetic fatty rats, are exposed to the hypoxic state in vivo or ex vivo [19–21]. In these models, islets are exposed to high oxygen consumption due to GSIS induced by high blood glucose levels, leading to hypoxia in islets. On the contrary, our study demonstrated that reduced vasculature in pancreatic beta cells led to reduced beta cell mass and function in VE-PDPK1-KO mice.

The integrity of the islet microvasculature is essential for normal islet function as it regulates the transport of nutrients and oxygen in addition to support of adequate paracrine interactions within each islet. However, since previous papers showed that a stronger reduction in vessel density does not always strongly affect islet function in vitro [22, 23], it is not clear whether the density alone is key to the phenotypes of our observation. Therefore, we evaluated capillary morphology by electron microscopy. We elucidated that capillary morphology was markedly altered in our endothelial cell-specific *Pdpk1*-knockout mice. We think that such alteration in capillary morphology in addition to the reduction of vessel density could, at least in part, explain the deterioration of islet function.

It was reported that beta cell-specific *Vegfa* knockout results in glucose intolerance and diabetes [24]. *Vegfa*-knockout mice displayed decreased density of the microvasculature and abnormal morphology of capillaries was observed. The pancreatic endothelial cells of the knockout mice had a thicker cell body and there were few fenestrae and thus the vessel permeability within islets was impaired. Interestingly, a recent report elucidated that overexpression of *Vegfa* in beta cells actually increased endothelial cell number but impaired islet morphogenesis and both alpha and beta cell proliferation [25]. These studies suggest that the precise control of *Vegfa* production by developing islet cells is important for normal islet development and vascularisation. In our model, the expression level of *Vegfa* was significantly increased in VE-PDPK1-KO mice, probably due to increased *Hif1α* expression. However, *PDPK1* knockdown in human umbilical arterial endothelial cells (HUAECs) revealed significantly reduced expression levels of vascular endothelial growth factor receptor 2 (VEGFR2) and this was not observed following *IRS2* knockdown (data not shown). It is possible that increased *Vegfa* could not increase islet vascularity due to reduced VEGFR2 expression in endothelial cells of VE-PDPK1-KO mice.

Electron microscopy observation revealed that cells were aligned along the basal membrane in islets of control flox mice (Fig. 5a). However, we cannot completely rule out that these remaining cells might be pericytes and not endothelial cells. In addition, basement membrane structures with no endothelium, so-called ‘empty sleeves’, were observed in islets of VE-PDPK1-KO mice (Fig. 5b). Nikolova et al reported that pancreatic beta cells do not form a basement membrane by themselves and that they attract endothelial cells and form capillaries with a vascular basement membrane next to pancreatic beta cells by using *Vegfa* [26]. We assume that endothelial cells were present at an earlier stage of development but left the islets of knockout mice due to lack of PDPK1, as basal membrane structures did exist in islets of both control *flox* and knockout mice. However, in the context of the report by Nikolova et al, the possibility that endothelial cells were removed during the procedure of digestion for islet isolation cannot be completely discounted.

VE-PDPK1-KO mice presented with reduced alpha and beta cell masses and impaired GSIS and response to Ex-4 in isolated islets. These results were not observed in endothelial cell-specific insulin receptor knockout (EndoIRKO) and endothelial cell-specific *Irs2*-knockout (ETIRS2KO) mice. There are some hypotheses which might explain the difference in phenotype between our VE-PDPK1-KO mice and these mice. First, VE-PDPK1-KO mice displayed decreased pancreatic blood flow and significantly increased ratio of pimonidazole-positive islets. It might be surprising that the ratio of pimonidazole-positive islets reached approximately 15–20% in control *flox* mice. However, this result was compatible with the findings in previous reports [27, 28]. Although speculative, we assume that hypoxia is induced to some extent even under normal conditions through some unknown mechanism. In addition, we cannot deny the possibility that pimonidazole shows false positivity under some experimental conditions. In ETIRS2KO mice, vascularity in islets was significantly decreased, while there was no difference in pancreatic blood flow and ratio of pimonidazole-positive islets. This means that islets in VE-PDPK1-KO mice were in a more hypoxic state compared with ETIRS2KO mice. It was reported that moderately hypoxic MIN6 cells presented similar features to VE-PDPK1-KO mice, such as decreased expression of insulin and its transcription factors such as *Mafa*, *Pdx1* and *Neurod* and *Glp1r* and impaired GSIS accompanied by activation of ER stress-related apoptotic genes [29]. There might be a threshold of hypoxia level, which determines beta cell survival rate in vivo and which needs further investigation. Second, insulin delivery to pancreatic islets through endothelial cells might be more severely impaired in VE-PDPK1-KO mice than in EndoIRKO and ETIRS2KO mice. It was reported that the PI3K signal plays an important role in transendothelial insulin delivery [30]. PDPK1 is the downstream molecule of PI3K. On the contrary, the insulin receptor could be compensated for by *Igf1r* and *Irs2* could be compensated for by *Irs1*, as both the insulin receptors and *Irs2* function upstream of PI3K. Therefore, it is possible that the mass and function of islets in VE-PDPK1-KO mice were more severely deteriorated than those in EndoIRKO and ETIRS2KO mice. Last, as described above, *PDPK1* knockdown in HUAECs caused an apparent decrease in VEGFR2 expression and its phosphorylation, not observed following *IRS2* knockdown (data not shown). VEGFR2 plays a very important role in normal microvascular formation including its fenestration. This might partially explain the difference between VE-PDPK1-KO and ETIRS2KO mice.

In conclusion, vascular endothelial *Pdpk1* plays an important role in the maintenance of pancreatic beta cell mass and function. Furthermore, our study demonstrates the different roles in vascular endothelial cells for *Pdpk1*, *Insr* and *Irs2*, all of these genes being important in insulin–PI3K signalling.

## Electronic supplementary material

ESM (PDF 386 kb)

## Data Availability

The datasets generated and/or analysed during the current study are available from the corresponding author on reasonable request.

## Abbreviations

EndoIRKO: Endothelial cell-specific insulin receptor knockout
ETIRS2KO: Endothelial cell-specific *Irs2-*knockout
Ex-4: Exendin-4
GLP-1: Glucagon-like peptide-1
GSIS: Glucose-stimulated insulin secretion
HBSS: Hanks’ Balanced Salt Solution
HUAEC: Human umbilical arterial endothelial cells
PDPK1: 3′-Phosphoinositide-dependent protein kinase-1
PI3K: Phosphoinositide 3-kinase
UPR: Unfolded protein response
VEGFR2: Vascular endothelial growth factor receptor 2
VE-PDPK1-KO: Vascular endothelial cell-specific *Pdpk1*-knockout
