# Supplementary material for: Vascular endothelial PDPK1 plays a pivotal role in the maintenance of pancreatic beta cell mass and function in adult male mice
Source: Diabetologia. 2019 May 4;62(7):1225–36. doi: 10.1007/s00125-019-4878-1 (PMC6560212; doi:10.1007/s00125-019-4878-1)
Supplement: Supplementary file 1 — (PDF 386 kb) [file 125_2019_4878_MOESM1_ESM.pdf]

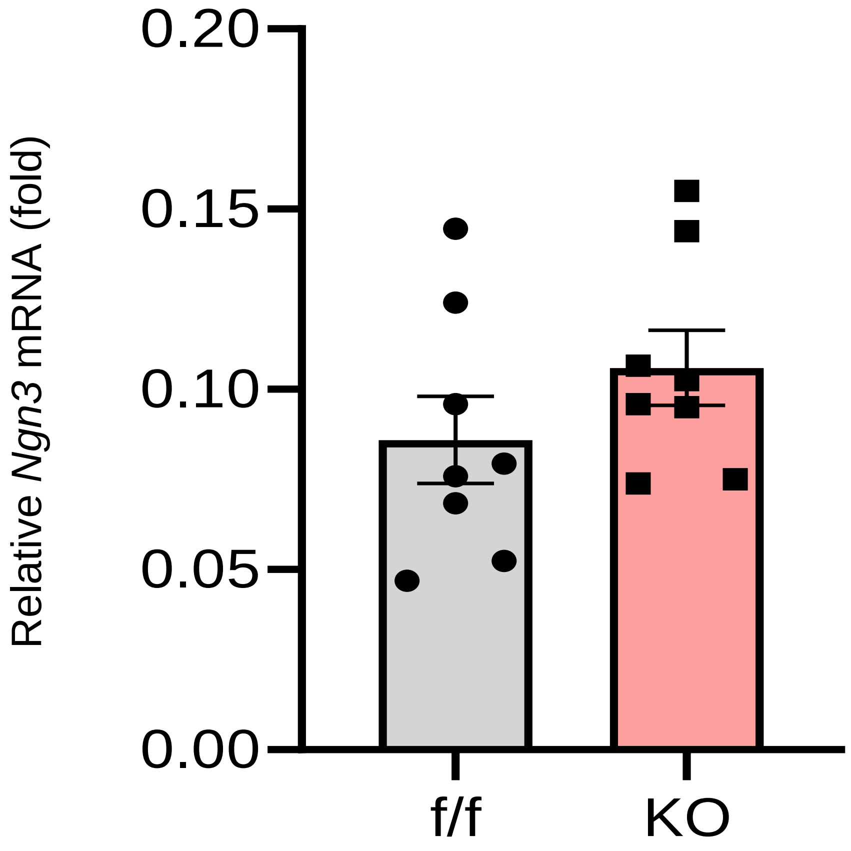

ESM Fig.1

Ngn3 mRNA expression in islets was comparable between control *flox* and VE-PDPK1-KO mice. (n=8 mice) Values are the means ± SEM. f/f, control *flox* mice; KO, VE-PDPK1-KO mice

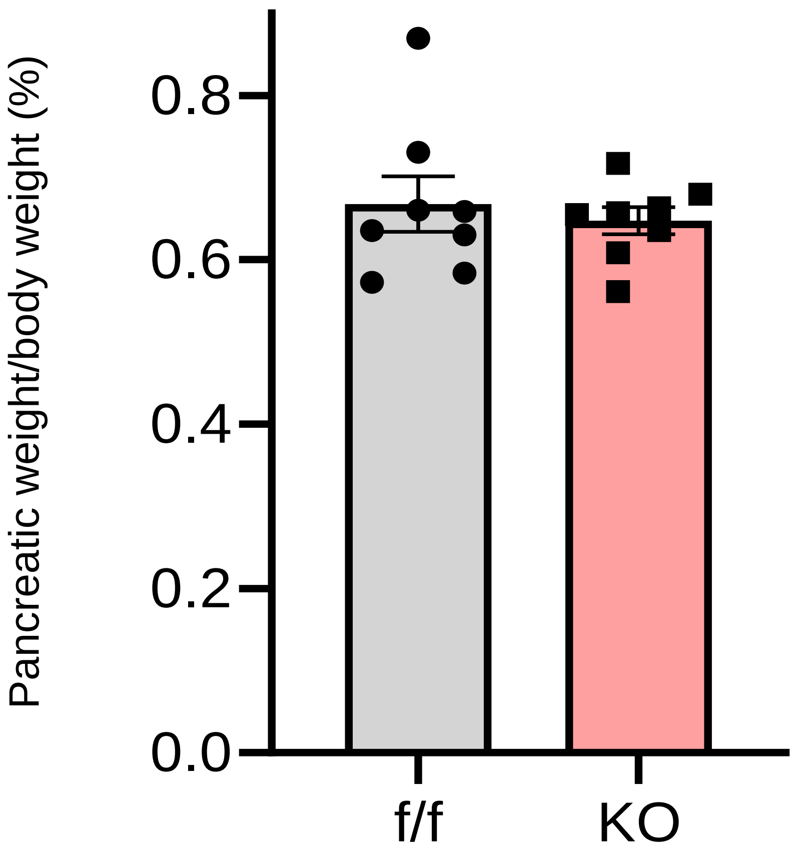

ESM Fig.2
There was no difference in pancreatic weight between control *flox* and VE-PDPK1-KO mice. (n=8 mice) Values are the means ± SEM. f/f, control *flox* mice; KO, VE-PDPK1-KO mice

ESM Fig.3

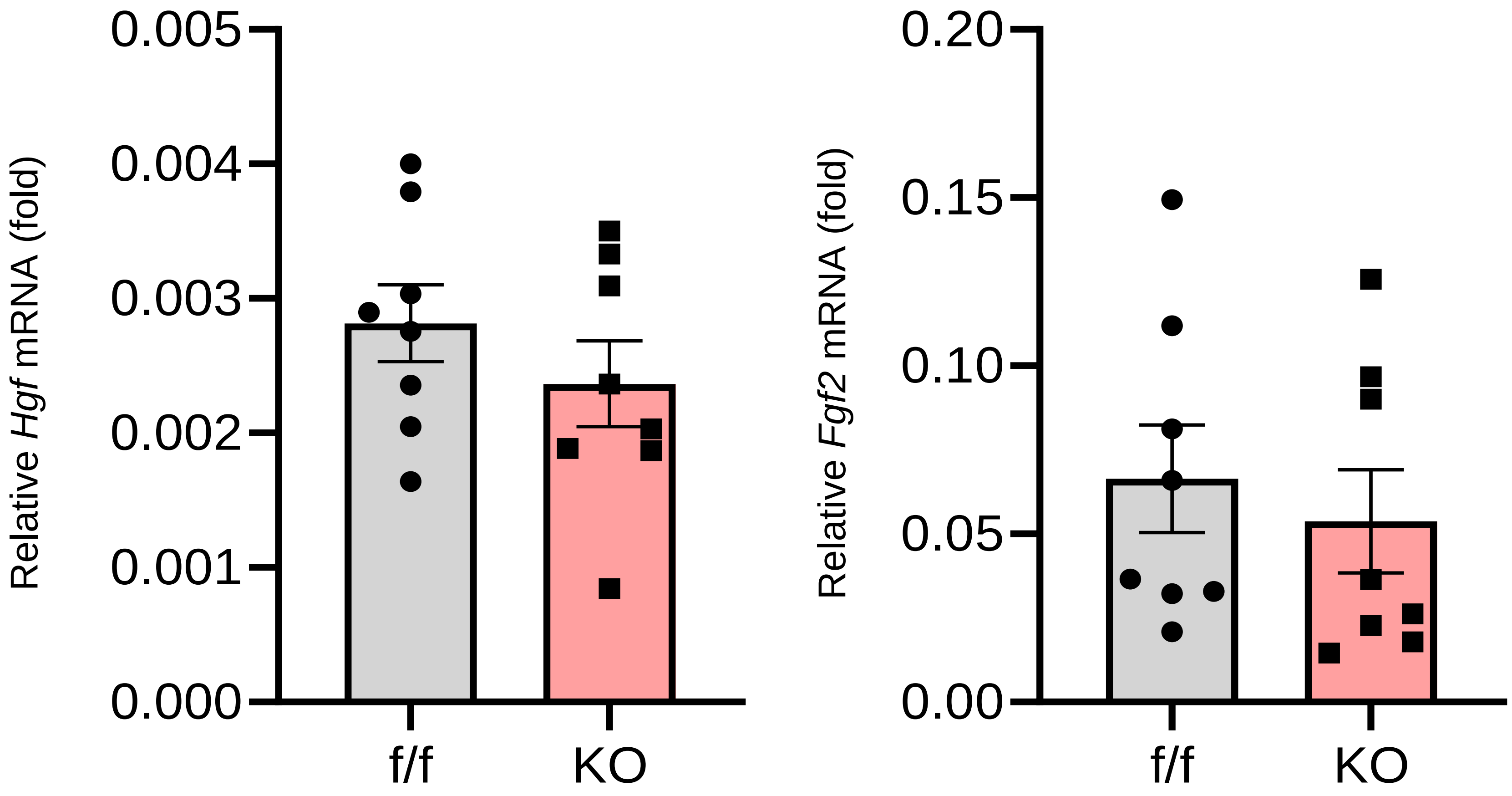

ESM Fig.3
mRNA expression levels of *Hgf* and *Fgf2* in islets were comparable between control *flox* and VE-PDKPK1-KO mice. (n=8 mice) Values are the means ± SEM. f/f, control *flox* mice; KO, VE-PDPK1-KO mice

Primer Sequences

	Forward	Reverse
mouse *Ins1*	CCCTTAGTGACCAGCTATAATCAGAGA	ACCACAAAGATGCTGTTTGACAA
mouse *Ins2*	CTGCTGGCCCTGCTCTTC	AACCACAAAGGTGCTGCTTGA
mouse *Mafa*	CCAGCTGGTATCCATGTCC	TTCTGTTTCAGTCGGATGACC
mouse *Pdx1*	CGGCTGAGCAAGCTAAGGTT	TGGAAGAAGCGCTCTCTTTGA
mouse *Neurod*	AGGAACACGAGGCAGACAAGA	CTCCCCCGTTTCTCAGAGAGT
mouse *Gck*	CCCTGAGTGGCTTACAGTTC	ACTGATGTGAGTGTTGAAGC
mouse *Slc2a2*	TTACCGACAGCCATCCT	TGAAAAATGCTGGTTGAATAGTAAAA
mouse *Glp1r*	ACTTTCTTTCTCCGCCTTGGT	CCTGGTGCAGTGCAAGTGTCT
mouse *Gipr*	GGAGCGCAACGAAGTCAAA	CTGGCCCTACCAAGATGGTTAT
mouse *Bcl2*	CGCTGCGGTGCTCTTGA	TCACACTCCGGCTTCACTGA
mouse *Bax*	GGAAGGCCTCCTCTCCTACTTC	TGAGGACTCCAGCCACAAAGA
mouse *Caspase8*	GAGATCCTGTGAATGGAACCTGGTA	GTTCACGCCAGTCAGGATGCTA
mouse *Caspase3*	CTGGACTGTGGCATTGAGACA	CAGCCTCCACCGGTATCTTC
mouse *Ccnd1*	TCGTGGCCTCTAAGATGAAGGA	CCTCGGGCCGGATAGAGTT
mouse *Irs2*	GGACCCCACCTGACTTCTCA	GCAGGAACAGGGAGCCTTATAA
mouse *Insr*	TGAAGGCAATGCCAAGGATATC	AAGACTGGCTGACTCATTGACAGT
mouse *Igf1r*	CCGCGCCAGTTTTGATG	AGGCAAGGCCCTCTCGTT
mouse *Irs1*	CGGGCTGACTCCAAGAACA	TCGCTATCCGCGGCAAT
mouse *Hif1α*	AGGAGCCTGATGCTCTCACTCT	TGTGTCATCGCTGCCAAAAT
mouse *Adm*	GCAATGCTTGTTGTCCAGCC	ACACACACACACACACACGGAAC
mouse *Eno1*	GATGGACGGCACAGAGAATAAATC	AGGCAGGATGACTTCAGGGTTG
mouse *Tpi1*	CCTTCCATTGGTTTGGGCTG	AATACAGGGGCTTTGGCACC
mouse *Hmox1*	CCACACAGCACTATGTAAAGCGTC	GTTCGGGAAGGTAAAAAAAGCC
mouse *Vegfa*	GTACCTCCACCATGCCAAGT	GCATTCACATCTGCTGTGCT
mouse *Bip*	TCATCGGACGCACTTGGAA	AACCACCTTGAATGGCAAGAA
mouse *Fkbp11*	ACACGCTCCACATACACTACACGG	ATGACTGCTCTTCGCTTCTCTCCC
mouse *sXbp1*	GAGTCCGCAGCAGGTG	GTGTCAGAGTCCATGGGA
mouse *tXbp1*	TTCATGAATGGCCCTTAGCAT	AAAACAAGCCCCCTCAGGTT
mouse *Atf3*	CCTCAGAAGTCAGTGCGACC	CATCCGATGGCAGAGGTGTT
mouse *Ddit3*	GGAGCCAGGGCCAACAG	GCCATAGAACTCTGACTGGAATCTG
mouse *Trb3*	TCTTCAGCAACTGTGAGAGGACG	TCCAGACATCAGCCGCTTTG
mouse *Ngn3*	GCGCAAGAAGGCCAATGA	CAGCGCCGAGTTGAGGTT
mouse *Hgf*	TAAAACGTGCGCTCACAGTG	TTCTCCTTGGCCTTGAATGC
mouse *Fgf2*	TGTGGCACTGAAACGAACTG	AACAGTATGGCCTTCTGTCCAG
